# The Unmet Rehabilitation Needs in an Inclusion Health Integrated Care Programme for Homeless Adults in Dublin, Ireland

**DOI:** 10.3390/ijerph18157917

**Published:** 2021-07-27

**Authors:** Áine Carroll, Siobhan O’Brien, Dee Harrington, Clíona Ní Cheallaigh, Ann-Marie Lawlee, Prasanth Sukumar

**Affiliations:** 1 School of Medicine, University College Dublin, D04 V1W8 Dublin, Ireland; siobhan.obrien@ucdconnect.ie (S.O.); prasanth.sukumar@ucd.ie (P.S.); 2 National Rehabilitation Hospital, A96 E2H2 Dublin, Ireland; dee.harr@hotmail.com; 3 St James’s Hospital, D08 NHY1 Dublin, Ireland; nicheacm@tcd.ie (C.N.C.); alawlee@stjames.ie (A.-M.L.); 4 School of Medicine, Trinity College, D02 PN40 Dublin, Ireland

**Keywords:** inclusion health, homelessness, disability, rehabilitation, integrated care, acquired brain injury, addiction, musculoskeletal

## Abstract

Background: People who become homeless have higher morbidity and mortality, use a disproportionate amount of healthcare resources, and generate a large volume of potentially preventable healthcare and other costs compared to more privileged individuals. Although access to rehabilitation is a human right under article 26 of the United Nations Convention on the Rights of Persons with Disabilities, the rehabilitation needs of individuals with homelessness have not been explored, and this project’s purpose was to establish a baseline of need for this cohort. Methods: A prospective audit of case discussions at an inclusion health service over a 2-month period in 2018. Results: Four multidisciplinary inclusion health clinics were observed with over 20 cases discussed in each and data were extracted using a bespoke audit data extraction tool. The inclusion health needs were diverse and complex with many unmet rehabilitation needs. Physical and cognitive rehabilitation needs were identified in over 50% of cases discussed. Musculoskeletal problems and acquired brain injuries were the most common cause of activity limitation. Most had concurrent medical conditions and addiction and/or mental health needs. None had access to rehabilitation services. Conclusion: The results of this study show that the rehabilitative needs of this cohort are significant and are not being met through traditional models of care. We are currently exploring innovative ways to provide appropriate services to these individuals.

## 1. Introduction

People experiencing homelessness have some of the highest and costliest health needs in a local community, but those needs are often overlooked when healthcare and social care services are planned and commissioned. There is a lack of comprehensive data on the health and care needs of the homeless with the National census in Ireland showing that the prevalence of disability is 27% in the homeless versus 13.5% in the entire population [1,2]. Since 2013, the number of homeless adults in Dublin has doubled [3]. People experiencing homelessness and other socially excluded individuals, for example people who inject drugs, prisoners, travelers, and undocumented migrants, have higher morbidity and mortality, use a disproportionate amount of healthcare resources, and generate a large volume of potentially preventable healthcare and other costs compared to more privileged individuals [4,5,6]. Although there is as yet no agreed conceptual framework for Inclusion Health, it is generally considered an emergent approach that aims to prevent and redress health and social inequities among the most vulnerable and excluded populations through service provision, research, and advocacy [7]. According to the World Health Organization, disability is the umbrella term for impairments, activity limitations, and participation restrictions, referring to the negative aspects of the interaction between an individual (with a health condition) and that individual’s contextual factors (environmental and personal factors) [8]. Many studies have reported that the prevalence of mental illness and physical and cognitive disability is higher among homeless individuals compared to the general population [9,10,11,12]. According to article 26 of the United Nations Convention on the Rights of Persons with Disabilities (UNCRPD), which has been ratified and signed by the Irish government, all persons with a disability should have access to habilitation and rehabilitation [13].

An Inclusion Health Integrated Care Program for homeless adults was developed in Dublin, starting with a pilot team consisting of a clinical nurse manager (A-ML) and consultant physician (CNiC) in August 2016. An iterative process was used to develop an integrated, multi-agency approach to managing cases of homeless adults with complex medical needs. As part of the program, a weekly interagency meeting was established, which was hosted by a day service for homeless adults (Merchants Quay Ireland). Health or social care providers working with homeless adults were invited to attend. Information about the meetings was spread informally through professional networks. The meetings were under the governance of St James’s Hospital, Ireland. Participants were invited to identify and discuss cases of homeless adults with complex medical and/or social needs. The individuals had given their consent to be discussed at the weekly meetings. As the service developed through an emergent process, the complexity of client needs became apparent. In particular, in team discussions, we recognized that the rehabilitation needs of these individuals had not been explored previously.

A desktop review of the literature using Google Scholar failed to reveal any empirical studies on specialist rehabilitation and homelessness. A search of the two highest impact rehabilitation journals, Clinical Rehabilitation and Archives of Physical Medicine and Rehabilitation, failed to reveal any papers addressing multidisciplinary rehabilitation and homelessness. Any publications that were available were discipline specific and not a holistic multidisciplinary approach. However, discipline-specific papers do show that clients benefit (increase employment and education prospects, money management, coping skills, and leisure activities) but also the challenges of engagement with such programmes [14,15,16,17]. In addition, AC contacted Rehabilitation Medicine Professor colleagues in other jurisdictions to assess the pathways of rehabilitative care available for homeless patients. There were none.

The purpose of this study was to utilize the weekly meetings to try and establish a baseline of need for this cohort without creating an unnecessary additional administrative burden on the multidisciplinary team (MDT).

## 2. Methods

As we sought to gain an initial insight into the unmet rehabilitation needs of clients attending the service without adding to the administrative burden and workload of participants, as a first step in understanding the problem, we chose to perform a prospective clinical audit. Clinical audit is defined as a quality improvement process that seeks to improve patient care and outcomes through a systematic review of care against explicit criteria and the implementation of change [18]. Prospective audit is based on the collection of information about patients during their process of care. It permits more reliable and complete clinical data collection, since the data required is predefined and can be validated and errors corrected while the data collection is in progress.

As there are no clinical standards for access to rehabilitation, we took a pragmatic approach using the UNCRPD as a guide. We developed a bespoke structured audit tool, which was used at the weekly multidisciplinary team (MDT) meetings. As this was the subject of a summer student research project, the data collection ran for 2 months over the summer in 2018. The MDT meetings were weekly case discussions of clients attending an Inclusion Health Service in an inner city homeless and drugs service. Participants in the meetings included an Inclusion Health Consultant (CNiC), a Rehabilitation Medicine Consultant (AC), Inclusion Health Nurse (A-ML), social workers, community health workers, nurses, and General Practitioners (GPs). Data were collected by one of the authors (SOB) who was a medical student attached to another author (AC). As such, she was known to the team members as she attended with AC, but she was not an active participant in team discussions. An initial audit data extraction tool was co-designed and piloted at 1 MDT meeting and amended iteratively as the complexity of the case discussions became apparent and the need for additional data fields emerged. By keeping headings broad, we felt that we captured the main challenges experienced without running the risk of potential identification. The headings chosen by the MDT included the following: demographic details (gender and age); medical diagnoses; presence or absence of rehabilitation needs; physical impairment; cognitive impairment; addiction challenges and/or mental health issues; and access to rehabilitation. These headings were entered into an excel spreadsheet and the data were collected in real time during the MDT meetings (SOB) with validation after the meetings with CNiC and AC. Purposive sampling was used as the issues and individuals discussed at the meetings were felt by the MDT to be representative of the issues experienced by the high complexity homeless population as a whole, and the timing of the study and the data collection was felt to be inclusive. All cases discussed at each of the MDT meetings were included.

A simple coding manual was created, which assigned a simple numeric code for all the possible answers to each heading in the dataset. A simple numeric code was assigned for the potential answers to each heading: 1 = yes, 2 = no. We adopted “9” as the universal code for missing data as recommended by the Health Services Executive Healthcare Audit Criteria and Guidance [19]

SPSS version 26 (SPSS Inc., Chicago, IL, USA) was used for descriptive and statistical analysis. Chi-square test was used to determine whether significant differences exist between groups in rehabilitation requirements.

## 3. Results

**Figure 1:**
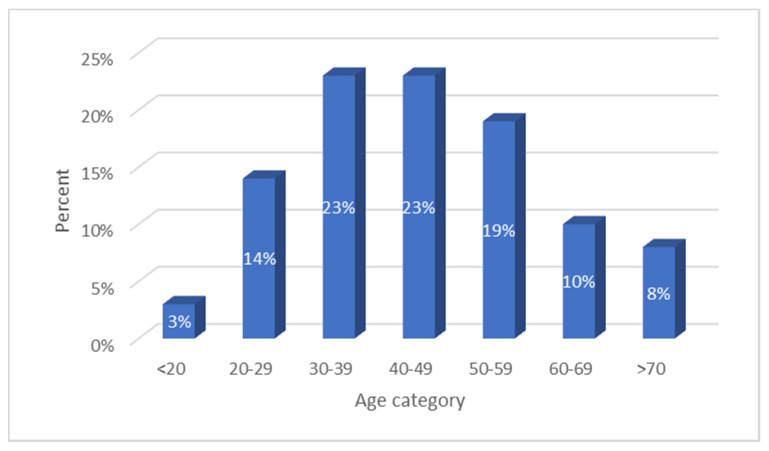
Age distribution of cases discussed.

There were four MDT clinics held over the period of the study. At each of the four clinics, over 20 cases were discussed with 97 cases being discussed in total. Of the cases discussed, 66 (67%) were male and 32 (33%) were female, while 70 (71%) cases discussed were under the age of 60 with the age distribution of cases shown in Figure 1.

**Figure 2:**
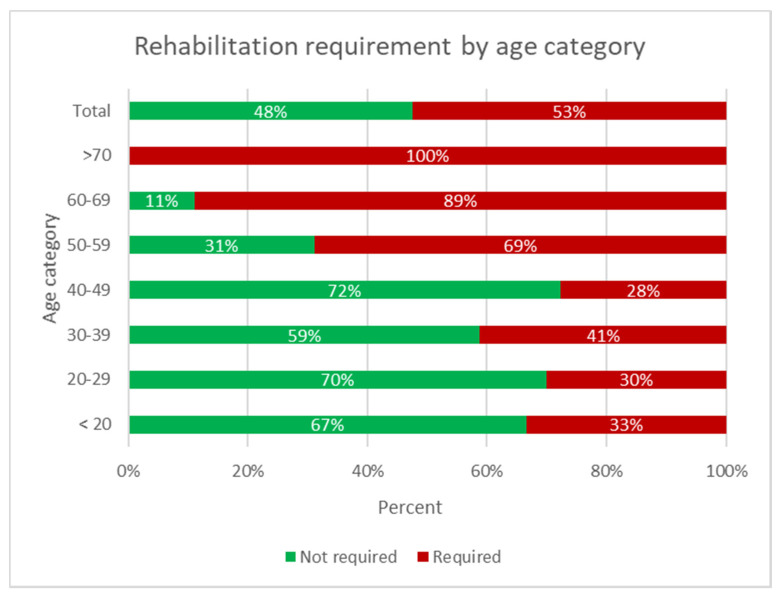
Rehabilitation requirements by age group.

In four cases, the underlying medical condition was not discussed. In total, 92 (96%) of cases discussed had complex medical histories, including multimorbidity, challenging social situations, and challenging behaviors. Meanwhile, 71 (72%) of cases had underlying chronic medical conditions and 49 (50%) had unmet rehabilitation needs as identified by the team. There was no statistically significant difference in gender and rehabilitation requirement (54% in males and 46% in females). There was a statistically significant difference by age group as shown in Figure 2, with χ^2^ of 20.564, 6 degrees of freedom, and *p* = 0.002.

**Figure 3:**
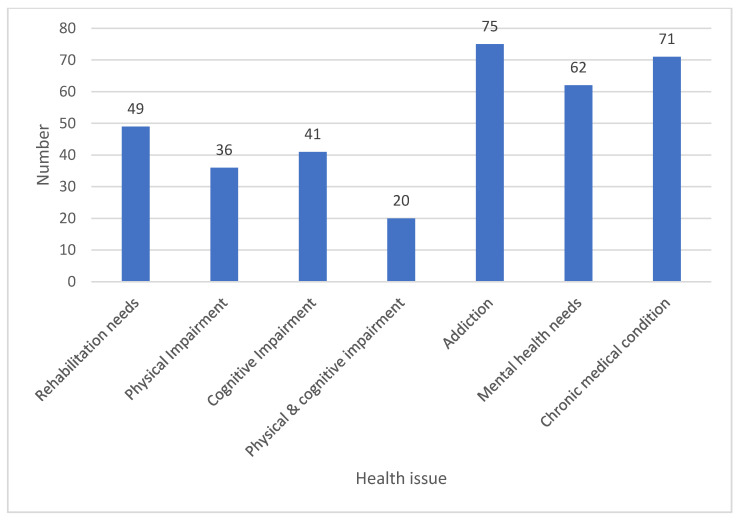
Needs identified in cases discussed.

In total, 36 (37%) had physical impairments and 41(42%) had cognitive impairments reported by the providers involved in their care, but only 20 (20%) had both physical and cognitive impairment. Seventy-five (76%) had addiction disorders, and 62 (63%) had mental health needs. These findings are summarized in Figure 3. Rehabilitation requirements were found to be highest among the physically and cognitively impaired.

## 4. Discussion

Although the team had an expectation of unmet needs in this population of clients attending an inclusion health service, we were all surprised at the extent and complexity of the needs. The cases discussed were mostly males under the age of 60 with underlying chronic medical conditions, and most had addiction and mental health challenges; physical and cognitive impairments were common, and unmet rehabilitation needs were present in 50%. Rehabilitation requirements were highest among those individuals with physical and cognitive impairment. The three cases that were under 18 years all had autism and intellectual disability.

As this was a prospective audit, a detailed understanding of the specifics of the rehabilitative and other health and care needs of the clients was not possible. We were also not able to assess if individuals had access to community-based general rehabilitation services, but it was the expressed view of the MDT that a traditional rehabilitation program would not suit the chaotic lifestyles of these individuals and that therefore, they would not have access to these services. Referral to complex specialist rehabilitation services requires a referral letter from a General Practitioner (GP) and will only be accepted with full contact details. Most of these individuals did not have a named GP or fixed address. In addition, a referral would trigger a multidisciplinary outpatient appointment at the complex specialist rehabilitation center, which is located in a rural part of Dublin, requiring transport. It was the view of the MDT that it is unlikely that such individuals would prioritize attendance, which is a view that is supported in the literature. It is recognized that people experiencing homelessness utilize healthcare in a different pattern to those that have a home, using primary care less, and use emergency departments more [20,21,22].

The research team had endeavored to locate a needs assessment tool that would cover the complexity of needs in this population but failed to identify an appropriate tool despite significant effort. A recent systematic review has confirmed the complexities of assessing, representing, and addressing the poor health of homeless populations, but this review did not consider rehabilitation needs [23]. This review recommended the development and consistent use of a suite of measures informed by and validated for people experiencing homelessness. We would agree with that assessment; however, we would be of the view that any tool should be co-designed with the people it is designed to serve.

This is a small prospective audit that was performed with very limited resources. We had limited access to advice on audit design and analysis; however, with good interdisciplinary working, we made best use of the resources that were available to us. Another challenge we faced was in deciding with whom the results should be shared. With health and social care services being siloed and fragmented, whilst many agencies may be interested in the findings, finding an integrated solution may not be straightforward.

We believe that there is a pressing need for better information on the health and care needs of people experiencing homelessness. This knowledge is required to inform effective resourcing, planning, and delivery of services by health and care organizations. Health and social care services are currently fragmented and siloed and are failing to meet the complex health and care needs of people experiencing homelessness.

## 5. Conclusions

This prospective audit has provided a first glimpse of the complex health and care needs of a cohort of persons experiencing homelessness attending an Inclusion Health Service. Although this study set out to get a sense of the unmet rehabilitation needs of this group, and we identified that 50% had unmet needs, the authors recognize that to explore these needs in isolation from the other significant needs of these individuals would reinforce the siloed nature of how health and care services are currently organized. The research team intends to build on the results of this study by co-designing, with persons experiencing homelessness, a comprehensive screening tool that reflects the complexity of health and care needs in this population. Access to rehabilitation is a human right under the UNCRPD, and person-centered integrated services are required if these individuals are to reach their full potential.

## Data Availability

Original Data is available from the lead author.
